# Study-based registers reduce waste in systematic reviewing: discussion and case report

**DOI:** 10.1186/s13643-019-1035-3

**Published:** 2019-05-30

**Authors:** Farhad Shokraneh, Clive E. Adams

**Affiliations:** Cochrane Schizophrenia Group, Division of Psychiatry and Applied Psychology, Institute of Mental Health, School of Medicine, University of Nottingham, Nottingham, UK

**Keywords:** Study-based registers, Grant application, Systematic reviews, Research prioritisation, Reducing waste, Increasing value

## Abstract

**Background:**

Maintained study-based registers (SBRs) have, at their core, study records linked to, potentially, multiple other records such as references, data sets, standard texts and full-text reports. Such registers can minimise and refine searching, de-duplicating, screening and acquisition of full texts. SBRs can facilitate new review titles/updates and, within seconds, inform the team about the potential workload of each task.

**Methods:**

We discuss the advantages/disadvantages of SBRs and report a case of how such a register was used to develop a successful grant application and deliver results—reducing considerable redundancy of effort.

**Results:**

SBRs saved time in question-setting and scoping and made rapid production of nine Cochrane systematic reviews possible.

**Conclusion:**

Whilst helping prioritise and conduct systematic reviews, SBRs improve quality. Those funding information specialists for literature reviewing could reasonably stipulate the resulting SBR to be delivered for dissemination and use beyond the life of the project.

## Background

### Time to complete systematic reviews

There is much redundancy in medical research [1–6] and systematic reviewing is no exception [7–18]. Usually, the review team runs searches, removes duplicates, screens titles and abstracts, obtains full-text reports, screens full texts, assembles reports of the same study, extracts data, synthesises them and writes the final report. This process has great potential for waste [19–21]. For systematic reviews, the median time from search to publication has improved from 14 months in 2008 [22] to 8 in 2013 [23] (mean time to complete was 17 months [24]; median time between first search and appearance of the review in PubMed was nearly 2 years [25]). The Cochrane Collaboration, a large organisation undertaking and maintaining systematic reviews of health care, largely works with volunteer health care professionals [26, 27] and the median time from Cochrane protocol to review publication was 2.4 years [28]. Keeping volunteer authors active on the review and the actual length of the review process are two major challenges to swift reviewing [29]. Efficiencies are needed.

### Current preparation for reviewing

At the start of a new systematic review or an update for an existing systematic review, there is limited knowledge about the quantity of relevant literature. Although estimation of workload is possible through piloting or scoping searches [30–32], this requires time and the exact number of relevant studies may remain unclear. This lack of clarity leaves assembled review teams vulnerable. The predicted investment of effort could be

- Overestimated—and eventually review teams have no or very few studies for their new review or update—with the waste this would incur.
- Underestimated—and the team is eventually surprised and, perhaps, overwhelmed with many relevant studies, with the risk of
  - ◦ Publishing a protocol but finding completion of the review unaffordable or impossible with the resulting wasteful unfinished or empty review.
  - ◦ Requesting extensions to funding; and/or
  - ◦ Running into delays that may render the final work being immediately out of date.
- Accurately estimated—but what remains unclear is as to whether the investment needed to review/update is warranted by any potential to change what is already known.

### Waste in systematic reviewing and information supply

The majority of the literature related to waste in systematic review are either focused on methodology [7–18] or automation of processes to shorten time-consuming tasks [33–36]. For over two decades, information specialists have given practical guidance for waste reduction in systematic reviews [32, 37–41]. Information specialists in the Cochrane Collaboration maintain specialised registers to support Cochrane reviews. Some of these registers are highly developed and shorten the systematic review process [42].

### Study-based registers

Study-based registers (SBRs) are databases in which all records of same study are linked to one ‘parent’ report. This study report may contain meta-data extracted from the various ‘child’ records of that same study. Often building a SBR involves an information specialist running searches across major bibliographic databases, de-duplicating, screening for eligibility, and obtaining full text of records. Then, there is the process of linking ‘child’ reports to the parent study record, extracting, cleaning and curating meta-data and maintaining the register with updates. In the case of randomised trials, meta-data for the study may be gleaned from the individual records (e.g. details of participants, interventions, controls and outcomes (PICO)) or, working from the other direction, from the overarching review in which the study has been used (e.g. qualitative or quantitative data incorporated within the review relating to that study). Details of *creating and maintaining* a SBR has been reported elsewhere [42].

## Aims and objectives

To describe how a SBR can be used to almost eliminate certain arduous steps in prospective systematic reviewing. We will illustrate how these steps can be accomplished in a matter of minutes or seconds and how this approach almost negates the early, inhibiting, and, we argue, wasteful, effort experienced by systematic reviewers. Although some benefits of SBRs have already been reported [42, 43], little has been presented on how SBRs can reduce waste whilst assisting prioritisation of systematic review work [44].

## ‘Living’ study-based registers

With a well-maintained SBR, an information specialist can provide the following data in a matter of minutes (stipulation of all estimates are review-specific but worked example follows):

- Exact number of
  - ◦ Studies/related records in a field (e.g. schizophrenia, tardive dyskinesia);
  - ◦ Studies/related records relevant to a new title or update (e.g. vitamin E for people with tardive dyskinesia);
  - ◦ Studies/related records relevant to a class of interventions (e.g. calcium channel blockers);
  - ◦ Studies that have/have not already been data-extracted, and the extracted data were available;
  - ◦ Existing related reviews on a topic—and quantification of studies/related records within each review;
  - ◦ Comparisons possible to accurately scope existing relevant evidence on a given topic—and quantification of studies/related records within each comparison [45];
- Alerts to
  - ◦ New studies, records to known studies and novel relevant treatments;
  - ◦ Research gaps in topic areas devoid of/with a dearth of evidence;
- For the studies
  - ◦ Concatenated importable references, the output of each study or all the relevant studies;
  - ◦ Full reports of each studies collected into a study folder;
  - ◦ Completed data extraction forms of studies where available.

Essentially, a SBR should be ‘living’. These living curated registers involve minimal analyses and are maintained by an information specialist (Table 1). Such registers have been produced by the Cochrane Dementia (ALIOS), Renal/Kidney (maintained within MeerKat), Pregnancy and Childbirth, Stroke (DORIS) and Schizophrenia (within MeerKat) teams for over two decades. For some existing SBRs, there is further developments to add functions to include extracted data from reviews [42], links to standard text and to prioritise sharing these data publicly [46]. Unfortunately, CENTRAL and Cochrane Register of Studies (CRS) are, at best, rudimentary SBRs at the time of revising this paper (27 March 2019).

**Table 1:**
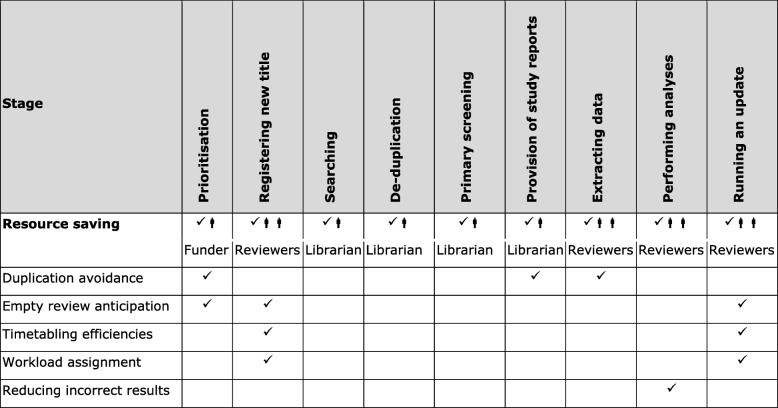
Saved resource by use of study-based registers by stage of systematic reviewing

Armed with the information from these sophisticated registers a potential review team should be able to present a much more accurate estimate of workload before embarking on the grant application or the actual review or update. These registers should make it possible to truncate the period immediately after protocol publication, seeding the systematic review with extracted data and preparing for swift meta-analysis.

## A case report from schizophrenia

Cochrane Schizophrenia has maintained a SBR of randomised trials for over two decades [47]. Routine searching identifies records that, with some help from automation, are merged into study reports (examples of studies with 10, 50 or even 100 records are not rare) helping minimise the risk of multiple counting with the systematic review. Meta-data (including number randomised) are part of the study record. Although increasingly automated, this process is facilitated by the group’s information specialist (FS). Since search strategies have been saved in bibliographic databases, monthly automatic updates are received through email. Then the information specialist spends three days per month for routine processes of updating the register: (1) 1 day for primary screening of search results and adding references to the register; (2) another day for obtaining full texts and linking them to their references; and finally (3) one last day for indexing the PICO meta-data from each full text and then assembling the separate references of the same study and linking them to that study. This register supports 324 maintained systematic reviews (17 May 2019).

**Fig. 1:**
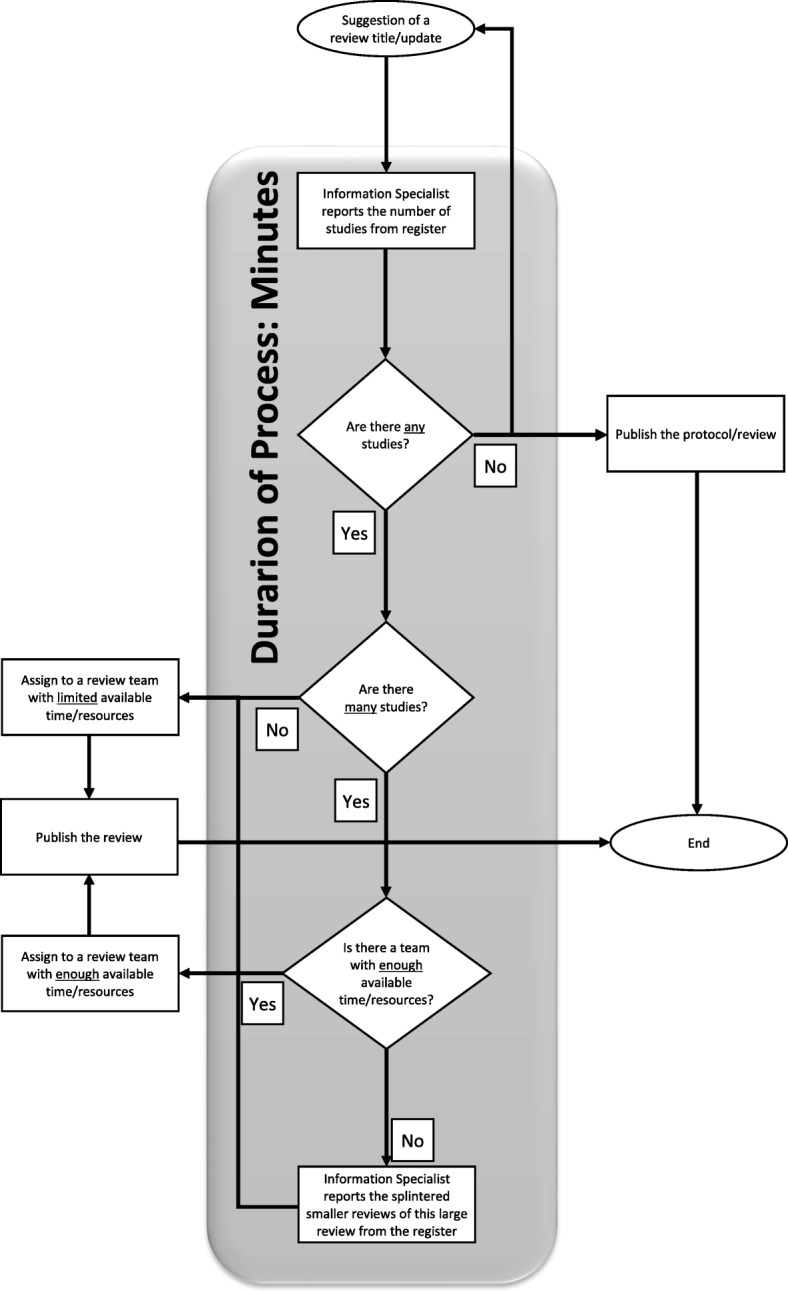
The process of systematic reviewing using a study-based register

Using this SBR, prioritisation of work could then proceed with efficiency (Fig. 1) and in line with items 2–6 from module 2 of SPARK, a prioritisation tool for systematic reviews [48].

### Estimates of costs for the grant application

In applying for NIHR UK Health Technology Assessment (HTA) Project Grant (14/27/02) [49–51], a call for reviews relevant to treating people with Tardive Dyskinesia (a problematic adverse effect of antipsychotic drugs) use of the SBR gave a clear advantage. Cochrane Schizophrenia’s information specialist ran a highly specific, highly sensitive search (16 July 2015) in the SBR and identified the exact number of studies relevant to the problem (time spent on task 8 s). This number helped the grant application team provide an accurate assessment of the work to be done—and realistic estimates of costs.

### Prediction of best composition of families of reviews

Tardive Dyskinesia is a condition for which many treatments have been used [52]. Arguments exist for ‘lumping and splitting’ at all sorts of levels. At the broadest level of ‘lumping’, the overview could encompass all treatments but this becomes unwieldy and impossible to update. At the finest level of ‘splitting’ each individual comparison of each treatment could be treated as a separate review. Even in a limited topic area such as Tardive Dyskinesia, this would lead to hundreds of separate reviews. Clearly there is a balance to be struck. By use of a controlled vocabulary for the meta-data within the SBR auto-grouping into logical treatment/comparison families for reviews can take place—and, once established, this can take place instantly. This ensures a pragmatic middle road dividing work into clinically logical bite-size reviews for later overviewing if required. Also, the classification of interventions within the register allows reviewing a class of interventions in a review. In the case of Tardive Dyskinesia, 10 separate review groupings were created (Table 2) (time spent on task 2 min and 10 s). This also helped the grant application team provide an accurate assessment of the output the funders could expect.

**Table 2:** Updated/started Cochrane reviews as a result of NIHR HTA Grant (14/27/02) [49]

Anticholinergic medication for antipsychotic-induced tardive dyskinesia [53]	
Antipsychotic reduction and/or cessation and antipsychotics as specific treatments for tardive dyskinesia [54]	
Benzodiazepines for antipsychotic-induced tardive dyskinesia [55]	
Calcium channel blockers for antipsychotic-induced tardive dyskinesia [56]	
Cholinergic medication for antipsychotic-induced tardive dyskinesia [57]	
Gamma-aminobutyric acid agonists for antipsychotic-induced tardive dyskinesia [58]	
Miscellaneous treatments for antipsychotic-induced tardive dyskinesia [59]	
Non-antipsychotic catecholaminergic drugs for antipsychotic-induced tardive dyskinesia [60]	
Pyridoxal 5 phosphate for neuroleptic-induced tardive dyskinesia^a^ [61]	
Vesicular monoamine transporter inhibitors versus placebo for antipsychotic-induced tardive dyskinesia^b^ [62]	
Vitamin E for antipsychotic-induced tardive dyskinesia [63]	

^a^This review is absent in the published report [51] because there was no new study

^b^This review is absent in the published report [51] because we became informed and started this review as a result of update search process in SBR

### Prediction of effort needed at data extraction step and saving effort for others

In this particular case, the SBR also contains information on already extracted data. Therefore, the applicants were also informed of exactly how much work has been completed and allowed them to make accurate costing for the necessary remaining efforts (time spent on the task 8 s). Working with such a register affords applicants opportunities to ensure that their request for funding for this part of the effort can be seen as an investment. The extracted study data can, thereafter, be made available to anyone thus reducing future duplication of effort (see below).

### Supply of documents

SBR systems such as Microsoft Access ‘MeerKat’ [64–66] have capacity to output file batches grouped by review, sub-grouped into relevant study files, in turn containing all relevant records and references (time spent on the task 4 min and 43 s). In this cause, this allowed those applying for the grant to reassure funders that supply of documents was not an issue and, once the grant was given, to waste no time in acquiring papers and piecing together the studies from ‘salami’ or multiple publications of same study.

### Future supply of full dataset

In the hope of evolving SBR towards making the level of document supply described above redundant and saving more time in the future—applicants sought and were granted support to extract all data from all randomised studies relevant to Tardive Dyskinesia and to make these data publicly available. This included each part of the data being made traceable to the exact site within the source record [67]. Any new updates of this will involve supply of documents containing tabulated, reliably and verifiably extracted data [50].

### Updating

Cochrane recommends biennial update for reviews [68] but this timing is not always appropriate. Excessive updating wastes resource while inadequate updating could result in outdated or incomplete evidence being used [69]. While there are methods to detect if updating a review could change the current conclusion/practice, almost all require an awareness of the available ‘unused’ relevant literature [48, 70–99], and some degree of screening and data checking to allow an informed decision. Within a well-constructed and maintained study register, this investment has already been made.

#### The upside

As the grant [49] was drawing to a close and the reviews were being completed. On the 26 April 2017, the SBR allowed the information specialist to run a final ‘just-before-submission’ update search limiting to not-already-identified records (time spent on the task 13 s). Just before publication, this search was used to inform the team that seven of the 10 reviews were fully current but two needed to be updated with a total of five new studies. This allowed the grant holders efficiently update the reviews just pre-publication to ensure they held fully current information.

#### The downside

This search also identified two new drugs (Valbenazine and Deutetrabenazine) entering the market specifically for treatment of people with Tardive Dyskinesia. These new compounds, unrelated to others, necessitate a new review outside of what was supported by the grant [62]. Unlike the decades ago when SBRs did not exist or were not sophisticated, it is now almost impossible to fail to identify a newly emerging treatment. This saves further waste in systematic reviews through inclusiveness of all treatments from all classes.

### Feasibility of study-based registers

Although it seems exciting to start a systematic review with the extraction of data, the workload creating a SBR should not be underestimated. The investment of time is a frequent concern. Is it possible for all evidence-synthesis groups to maintain a SBR and what are the necessary requirements in creating such a register?

The short answer is that every systematic review is, in itself, a small SBR. Frequently at completion of any given review, these small registers (reviews) are rendered unusable to others or disassembled necessitating the next interested group of reviewers to have to repeat the construction. This is avoidable waste when collating all the data within a related group of reviews constitutes the embryonic SBR.

In Table 3, we itemise the time and resource required for establishing and maintaining our broad-based schizophrenia SBR.

**Table 3:** Characteristics of the study-based database in this study

Volume	Records:	~ 20,000 studies ~ 30,000 references/reports
	PICO meta-data:	~ 230 healthcare conditions; ~ 2700 interventions^a^; ~ 13,700 outcomes
Variety	Standard protocols for meta-data:	For references (RIS); For studies (PICO)
Veracity	Document coverage	
	Type:	Any
	Language	All
	Date/time:	Any
	Geography	Worldwide
	Publication status:	Published/unpublished
	Status of study:	All^b^
	Reliability	
	Two independent Information Specialists checked data.	
Velocity	Information specialist	Screens 1000–2000 references per month; Adds 100–200 eligible references to register.
Value	Software: free.	
	Current number of maintained systematic reviews: 324.	
	Retracted studies: retraction linked into study record.	
	Reproducibility and replicability: all SBR’s review-specific steps can be repeated within seconds [100].	
	Prioritising: sensitive/specific direction of effort	
	Human resources: skilled information specialist	
	Establish register	1 year (F/T) 2–3 years (P/T 50%)
	Maintain register	1 day/week

^a^ Structured, controlled language (e.g. WHO ATC)

^b^ Finished/ongoing/awaiting/terminated/unclear

## Conclusions

Small SBRs, in the form of competed reviews, are increasingly prevalent. We maintain that there is a strong argument for creation of broad-based healthcare study-based registers linked to records containing data, text and other relevant information. Not to use already compiled data is wasteful and not to invest to create the SBR is passing cost—and waste—down the line to reviewers. Information specialist investment is already happening—repeatedly. We argue that focus and direction of this investment would avoid the ongoing unnecessary multiplication of effort [101].

We reported one example of the potential of SBRs for grant application. This is one amongst many. The living property of this register allowed the information specialist with his/her more sophisticated role—to become an integral—and useful—part of the review team.

Finally, the SBR promoted more sophisticated sharing of data from this project facilitating the not-so-distant full automation of living systematic reviews.

## Abbreviations

HTA: Health Technology Assessment
PICO: Participants/Problem/Population, Interventions, Control/Comparison, and Outcomes
RIS: Research information systems
SBR: Study-based register
SPARK: The centre for systematic reviews on health policy and systems research
